# Will Food Safety Incidents Stimulate the Public’s Desire for Food Safety Governance?

**DOI:** 10.3390/foods13223693

**Published:** 2024-11-20

**Authors:** Xixi Mao, Changlong Hao

**Affiliations:** 1 School of Marxism, Jiangnan University, Wuxi 214000, China; maoxixi@yeah.net; 2 State Key Laboratory of Food Science and Resources, Jiangnan University, Wuxi 214000, China; 3 School of Food Science and Technology, Jiangnan University, Wuxi 214000, China

**Keywords:** food safety incidents, event system theory, risk perception, risk communication, governance willingness

## Abstract

This paper, grounded in the Stimulus–Organism–Response (SOR) model and event system theory, examines the mechanisms through which public recognition—specifically novel, disruptive, and critical recognition—of the intensity of food safety incidents influences willingness to engage in food safety governance. Risk perception is identified as a mediating variable, while risk communication serves as a moderating variable. Based on survey data, the study found that various forms of public recognition significantly impact governance willingness. Furthermore, risk perception plays a crucial mediating role, and risk communication has a notable moderating effect on the relationship between risk perception and food safety governance willingness. These findings illuminate the intrinsic connection between public recognition of food safety incidents and governance willingness, offering robust theoretical support and practical guidance for enhancing food safety governance. This research contributes to the ongoing improvement and optimization of food safety governance systems.

## 1. Introduction

Food safety means ensuring that food does not pose a risk to consumer health throughout its production, processing, storage, transportation, and sale. Food safety is a foremost necessity for people, with safety as the priority. High-profile food safety incidents, such as the melamine milk powder scandal in 2008 and the more recent counterfeit Thai fragrant rice incident exposed during the 2024 CCTV (China Central Television) 3·15 evening gala, have significantly impacted consumer confidence and public health. These incidents, which include foodborne diseases and contamination, pose serious threats to human health and are considered major social risks in China [1,2,3,4,5,6,7]. These incidents not only severely compromise public health but also significantly impact trust in the food safety system, triggering public anxiety and concerns about food safety that spread through online public opinion [8,9]. Public opinion on food safety is heavily influenced by media coverage of these incidents. The rapid dissemination of information through traditional and social media channels often amplifies public anxiety and concern. For instance, the cancer risks of aspartame revealed by the International Agency for Research on Cancer and the mixing of edible oil in oil tank trucks have swiftly become focal points of public attention. This heightened risk perception can lead to a decline in consumer confidence and a demand for more stringent food safety governance.

China preliminarily established a risk regulation and co-governance model for food safety supervision centered on the ‘Food Safety Law’, which officially came into effect on 1 June 2009 and was subsequently revised in 2015, with the current effective version being the 2018 revision. And China has long implemented a government-led monopoly supervision model, where the government assumes the role of a manager in formulating laws and regulations and constructing and improving the governance system. However, the food safety incidents exposed in recent years have revealed the inadequacies of the current legal and regulatory frameworks. Due to the intertwined technical and social aspects of food safety issues, the government may face challenges in comprehensively addressing complex risks, potentially constraining the effective operation of market mechanisms [10,11]. In the face of repeated government and market failures in food safety supervision, social co-governance is seen as the key path to achieving modernization of food safety governance, emphasizing the collaborative participation of the government, enterprises, social organizations, and the public. In other words, researchers have highlighted the significance of public awareness in food safety governance. As important participants in food safety governance, the public are not only the actual beneficiaries but also the spectators of food safety incidents. Therefore, enhancing public food safety awareness and participation is of great importance for improving food safety governance [12,13,14]. Social forces, as an integral part of the social co-governance of food safety, are seen as an effective supplement to government governance and corporate self-discipline [15], providing grassroots information to enhance the legitimacy of interventions.

Promoting the public’s direct participation in food safety governance, as the most immediate stakeholders, is an important direction for mechanism innovation [14,16,17,18,19]. As an independent third party to market mechanisms and government regulation, the public participates in food safety governance, overcoming government and market failures in food safety regulation, and achieving good governance in food safety. The public, as an indispensable part of food safety co-governance, is driven not only by “interests” as the actual beneficiaries of food safety management but also by the “spectator” mentality driven by interest in food safety incidents. Therefore, it is necessary to construct and maintain a long-term public participation governance mechanism [20,21,22,23].

Although a substantial amount of research has proven that food safety incidents are the trigger points for changes in public willingness to participate in food safety governance, the mechanism of interaction between food safety incidents and public willingness to participate in food safety governance is not yet clear. Based on this, this paper focuses on three core issues: How do food safety incidents affect public risk perception? Does risk perception play a mediating role between the intensity of food safety incident cognition and governance willingness? How does risk communication regulate the impact of risk perception on governance willingness? To clarify these issues, this paper examines the impact mechanism of the public’s cognition of food safety incident intensity on their willingness to participate in food safety governance through a conditional process model, clarifying the mediating role of risk perception and the moderating role of risk communication and aiming to provide solid theoretical support and practical guidance for the government and enterprises to formulate more precise and effective food safety governance strategies. At the same time, this study helps to deepen public cognition of food safety issues and enhance the willingness to participate in food safety governance, thereby promoting the overall improvement of the entire society’s food safety governance level and advancing the continuous optimization and improvement of the food safety governance system.

## 2. Theoretical Framework and Research Hypotheses

**Figure 1:**
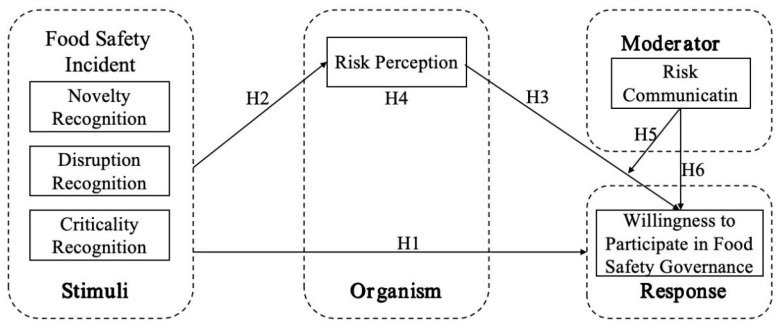
Theoretical framework.

This section outlines the theoretical framework guiding this study and presents the research hypotheses derived from it. To explore how public cognition of the intensity of food safety incidents influences their willingness to engage in food safety governance, this study integrates the Stimulus–Organism–Response (SOR) model with event system theory, incorporating elements of risk perception and risk communication to construct a conditional process model of the impact of food safety incidents on public engagement in governance (as illustrated in Figure 1).

On one hand, the SOR model provides a theoretical framework for understanding how external stimuli influence an individual’s internal psychological state, thereby guiding their ultimate behavioral response. According to this model, the public’s cognition of the intensity of food safety incidents serves as the stimulus (S), affecting the public’s risk perception as the organism (O), which in turn stimulates or inhibits the public’s willingness to engage in food safety governance (R). On the other hand, event system theory emphasizes the significant role of event characteristics in shaping individual cognition and behavioral responses. Generally speaking, the greater the novelty, disruption, and criticality of an event, the greater its impact [24]. Among these, event intensity, as one of the core elements of event system theory, directly reflects the severity and influence of an event. Given the difficulty in accurately quantifying or tracking the spread and impact of food safety incidents across different regions and time periods, this paper analyzes food safety incidents from the perspective of event intensity.

In the context of a mediated society, risk perception and risk communication are two closely intertwined and increasingly important issues. Risk perception is a psychological sense of uncertainty on the part of consumers, referring to their awareness of uncertainty or adverse and harmful outcomes during consumption. However, when applied to the study of consumer behavior following food safety incidents, it was found that consumers’ food purchasing behavior is influenced by risk perception, risk attitudes, and the interaction between the two [25]. Although risk perception affects purchasing decisions in the food safety consumption process, in reality, there is often a discrepancy between the actual food safety risks and consumers’ perceptions of these risks, with individual food safety consumption decisions being influenced more by subjective perceptions than by objective risks [26]. When food safety incidents occur, an increased level of food safety risk perception among consumers can significantly reduce the purchase of related foods [27]. The “ripple effect” of food safety incidents can elevate short-term risk perception, leading to a decline in confidence. To avoid risk, consumers often take preventive measures, such as reducing purchase quantities [28]. This is related to the important pathways through which information about food safety incidents circulates and spreads among the public; that is, the risk communication of food safety incidents not only conveys information but also indirectly affects the behavioral responses of the public by influencing public emotions and attitudes. Given the critical importance of risk communication in food safety issues and the rumors that often accompany them, the dissemination of false information can easily lead to social panic. Therefore, this paper also examines risk communication as a moderating variable to further explore the boundaries of how risk perception influences the public’s willingness to engage in food safety governance.

To investigate the relationships among the variables in this model, this study proposes the following specific hypotheses:

**H1:**  *Food safety incidents have a significant positive impact on food safety governance.*

**H1a:**  *Novel recognition of events has a significant positive impact on food safety governance.*

**H1b:**  *Disruptive recognition of events has a significant positive impact on food safety governance.*

**H1c:**  *Critical recognition of events significantly positively impacts food safety governance.*

**H2:**  *Food safety incidents have a significant positive impact on risk perception.*

**H2a:**  *Novel recognition of events significantly positively impacts risk perception.*

**H2b:**  *Disruptive recognition of events significantly positively impacts risk perception.*

**H2c:**  *Critical recognition of events significantly positively impacts risk perception.*

**H3:**  *Risk perception positively influences food safety governance.*

**H4:**  *Risk perception mediates the relationship between food safety incidents and food safety governance.*

**H4a:**  *Risk perception mediates the relationship between novel recognition of events and food safety governance.*

**H4b:**  *Risk perception mediates the relationship between disruptive recognition of events and food safety governance.*

**H4c:**  *Risk perception mediates the relationship between critical recognition of events and food safety governance.*

**H5:**  *Risk communication moderates the relationship between risk perception and food safety governance.*

**H6:**  *Risk communication positively impacts food safety governance.*

## 3. Research Methodology

### 3.1. Survey Design and Administration

This study employed a self-administered questionnaire, which was refined following expert review and a pilot survey. Questions with ambiguous semantics or ambiguous expressions were revised, and items with factor loadings below 0.6 were eliminated to form the final survey questions used for data collection. A combination of online and offline methods was utilized during the data collection phase to broaden the scope and enhance the diversity of the sample. Online surveys were primarily conducted through social media, email, and online survey platforms, offering wide coverage and convenience for respondents to participate at any time and place. Offline surveys were mainly conducted in public places such as shopping centers, supermarkets, and communities, collecting data through face-to-face interactions, which increased the reliability and richness of the data. As of 30 June 2024, a total of 750 questionnaires were distributed, and 738 were collected. After excluding questionnaires with significant invalid or missing data, a total of 716 valid questionnaires were obtained, with a recovery rate of 95.47%. The following analysis is based on the questionnaire survey data. To ensure the reliability of the survey data, in-depth interviews were conducted with some participants to further verify the authenticity of the questionnaire data and to deepen the comprehensive understanding of the public’s willingness to participate in food safety governance. The following analysis is strictly based on the data obtained from the questionnaire survey for in-depth discussion.

### 3.2. Descriptive Statistical Analysis of the Sample

The first section of the questionnaire gathered personal information from respondents, including gender, age, marital status, education level, occupation, family structure, and residential area (Table 1). These variables were used as control variables in the analytical model. The sample comprised demographic data from 716 participants.

In terms of gender distribution, the sample was relatively balanced, with females slightly outnumbering males at 52.23% and 47.77%, respectively. The age structure of the sample included a diverse range of age groups, with individuals aged 25 to 45—representing the young and middle-aged demographic—comprising over half of the participants (50.27%). Regarding marital status, the majority of respondents were married (52.23%), indicating that most participants have established, stable family relationships. In terms of education level, those with junior college and undergraduate degrees made up more than 40% of the sample (45.53%), while participants with high school, technical secondary school, or vocational school degrees accounted for a notable proportion (25.84%).

Concerning occupation, workers in the service industry and those in production and transportation represented the two largest occupational categories, accounting for 27.37% and 21.51% of the sample, respectively. In terms of family structure, nuclear families and extended families were the most prevalent, comprising 32.82% and 37.85% of the sample, while single-person households and single-parent families also represented a significant portion. Regarding family economic status, the sample encompassed a wide range of income levels, from less than 5000 yuan to over 20,000 yuan. Notably, families with a medium income level of 8000 to 14,999 yuan constituted nearly half of the participants (47.76%). Finally, concerning residential area, the majority of participants lived in residential communities and staff dormitories, accounting for 31.84% and 18.72%, respectively.

### 3.3. Variable Selection and Scale Design

The second part of the questionnaire, grounded in the aforementioned theoretical analysis and research hypotheses, focuses on six key variables: novel recognition, disruptive recognition, critical recognition, risk perception, risk communication, and willingness to participate in food safety governance. These variables form the core framework and analytical foundation of this study. In the questionnaire design process, the measurement of key variables draws on and refers to mature scales both domestically and internationally. To ensure the consistency of the measurement items, this study adheres to the “translation–back translation” principle; a statistics faculty member and a translation major faculty member were invited to conduct the preliminary translation of the scale. Subsequently, a food safety management professional was invited to revise it. Furthermore, before the formal survey, 50 questionnaires were distributed for a pilot study, and questions with unclear semantics or ambiguous options were modified to ensure the scientific design of the questionnaire and the accuracy of the scale translation. All scales use a 5-point Likert scoring method, requiring participants to rate each item from 1 (strongly disagree) to 5 (strongly agree).

Specifically, the measurement of event intensity cognition refers to the scale developed by Morgeson et al., and 10 items were designed in conjunction with typical food safety incidents in recent years [24]. The content covers three dimensions of event intensity, including the novelty of the event (4 items), disruption of the event (3 items), and criticality of the event (3 items). The measurement of risk perception integrates scales on food safety risk perception, with five items designed around the degree of perception, types of risks, and channels of information acquisition. The measurement of risk communication is based on the analysis from the perspective of risk communication, referring to the “social amplification of risk” framework proposed by Kasperson et al. [29], with a self-made scale designed to comprehensively assess the communication of food safety risks from multiple dimensions, such as communication channels, content quality, communication effects, communication strategies, and attention to special groups. The measurement of willingness to participate in food safety governance refers to the scale on food safety risk governance participation willingness, which measures the public’s willingness to participate in food safety governance from three aspects: attention willingness, governance willingness, and autonomy willingness.

## 4. Results Analysis

### 4.1. Reliability and Validity Analysis

This study utilized Amos 28.0 software to conduct reliability and validity assessments of the scales. To ensure the rigor of convergent validity for all constructs, confirmatory factor analysis (CFA) was performed.

Initially, it was identified that the DR4 (DR: disruptive recognition) item within event intensity, the CR4 (CR: critical recognition) item, and the RC4 (RC: risk communication) item did not meet the established minimum standard of 0.5 for factor loadings, leading to their exclusion from the analysis. Table 2 comprehensively presents the factor loadings of the remaining items, all of which fall within the recommended range, demonstrating satisfactory statistical properties. To further substantiate the scales’ reliability and validity, the average variance extracted (AVE) and composite reliability for each variable were calculated. On one hand, the CR values for all variables significantly exceeded the threshold of 0.7, a result that robustly attests to the high degree of internal consistency among the items measuring the same variable, indicating a high level of similarity and stability in their measurement of the same construct. On the other hand, the AVE values for all variables were reliably greater than 0.5, a finding that not only suggests the effective explanatory power of each variable over its corresponding items but also further confirms the good discriminant validity among variables, ensuring the rigor and reliability of the study’s conclusions.

Additionally, to assess the model fit of the proposed model, this study employed a variety of statistical indices for a comprehensive judgment. The χ2/df ratio of 1.547 is well below the commonly accepted threshold of 5, indicating a good overall model fit. The RMR (Root Mean Square Residual) value of 0.023 and the RMSEA (Root Mean Square Error of Approximation) value of 0.026 are both significantly less than the reference standard of 0.05, suggesting a low level of residuals and minimal model error. Furthermore, the GFI (Goodness-of-Fit Index) and AGFI (Adjusted Goodness-of-Fit Index) values are both 0.946, the CFI (Comparative Fit Index) is 0.956, the NFI (Normed Fit Index) is 0.958, and the TLI (Tucker–Lewis Index) is also 0.958; these indicators all exceed the excellent standard of 0.9, implying a high degree of consistency between the model and the observed data. Overall, the structural equation model proposed in this paper is statistically sound, with a good model fit, and it is suitable for further analysis.

The data in Table 3 reveal the uniqueness of each variable in the discriminant validity test and the complexity of their interrelationships. Specifically, the mean values of the variables, such as 2.25 for novel recognition (with a standard deviation of 0.95) and 6.29 for disruptive recognition (with a standard deviation of 0.88), not only reflect the distribution of participants’ attitudes across various dimensions but also demonstrate the dispersion of the data, further confirming the significant independence of these variables in measuring their distinct characteristics. At the same time, the correlation coefficients between variables, as key indicators of statistical association, clearly reveal the interrelationships among the variables. For instance, the correlation coefficient of 0.35 between novelty cognition and subversive cognition (indicating statistical significance) suggests a positive but not highly correlated relationship, indicating that while they are related in some respects, they each maintain unique measurement content. Similarly, the significant positive correlations between subversive cognition and criticality cognition (0.42), risk perception (0.38), and risk communication (0.45) reveal the synergistic effects of these core elements in fostering the formation of willingness to participate in food safety governance. It is noteworthy that risk perception (AVE square root of 0.81) and risk communication (AVE square root of 0.83), as mediator and moderator variables, not only validate their stability on their respective constructs with their high AVE values but also, through significant correlations with other variables, emphasize their bridging role between cognition and behavioral intentions.

The aforementioned analysis preliminarily revealed the interconnectivity among variables, which do not exist in isolation but are interwoven and collectively contribute to the formation and enhancement of willingness to participate in food safety governance. However, to ensure the reliability of the research hypotheses, especially those involving mediating and moderating effects, further statistical analysis were conducted for verification and are detailed in the following sections.

### 4.2. Hypothesis Testing

(1) The overall effect

The data in Table 4 support the research hypotheses regarding the positive impact of novel recognition, disruptive recognition, and critical recognition on risk perception and willingness to participate in food safety governance as well as the direct impact of risk perception and risk communication on willingness to participate in food safety governance. Additionally, this reveals the significant role of the interaction between risk perception and key variables in enhancing governance willingness.

The β coefficient for novel recognition on willingness to participate in food safety governance is 0.1672 (*p* = 0.0031), indicating a significant positive impact, thus validating research hypothesis H1a. The β coefficient for subversive cognition on willingness to participate in food safety governance is 0.0735 (*p* = 0.0714), which, while close to the significance level (*p* < 0.1), does not meet the conventional significance standard (*p* < 0.05). The direct positive impact of subversive cognition on governance willingness is weak but may still have a significant effect through other pathways (such as risk perception), thus indicating partial support for H1b. The β coefficient for criticality cognition on willingness to participate in food safety governance is 0.3451 (*p* < 0.001), demonstrating a significant positive impact with a substantial effect size, thereby confirming research hypothesis H1c.

The significant positive impact of food safety events on risk perception was not directly verified, but the significant impact of each cognitive dimension on risk perception can indirectly support this point. The β coefficient for novel recognition on risk perception is 0.1736 (*p* < 0.001), indicating a significant positive impact and validating research hypothesis H2a. The β coefficient for subversive cognition on risk perception is 0.2975 (*p* < 0.001), indicating a significant positive impact with a substantial effect size, thus confirming research hypothesis H2b. The β coefficient for criticality cognition on risk perception is 0.2094 (*p* = 0.0014), indicating a significant positive impact and validating research hypothesis H2c.

The β coefficient for risk perception on willingness to participate in food safety governance is 0.254 (*p* < 0.001), indicating a significant positive impact and thus confirming research hypothesis H3. Since novel recognition, disruptive recognition, and critical cognition of food safety events all have significant positive impacts on risk perception, and risk perception also has a significant positive impact on governance willingness, it can be indirectly inferred that risk perception mediates between food safety event cognition and food safety governance, thus validating research hypotheses H4a, H4b, and H4c. However, the specific magnitude of the mediating effect is not yet clear. Furthermore, the β coefficient for risk communication on willingness to participate in food safety governance is 0.125 (*p* = 0.0026), indicating a positive impact and thus confirming research hypothesis H6. Therefore, it can be concluded that effective risk communication strategies can enhance consumer participation and governance willingness, but the above analysis does not directly demonstrate the effect of risk communication as a moderating variable.

(2) Mediating Effect

To explore the mediating effect of risk perception, this study employed standardized effect values (β), Bootstrap methods (5000 resamples), and two-tailed significance tests for analysis. Table 5 shows that all mediating paths have significant statistical significance (confidence intervals do not contain zero), indicating that hypotheses H4a, H4b, and H4c are supported, proving that risk perception plays a crucial mediating role between different cognitive dimensions of food safety events and food safety governance.

Firstly, novel recognition not only significantly affects governance outcomes through direct paths but also produces a significant indirect effect through the mediator variable of risk perception. This dual influence mechanism works together on food safety governance, where the direct effect accounts for the main proportion (65.43%), and the indirect effect contributes to the remaining 34.57%. Secondly, subversive cognition is also an important driver of food safety governance. However, unlike novel recognition, the direct effect of subversive cognition, although significant, has a relatively small effect value. In contrast, the indirect effect through risk perception is more significant and accounts for a higher proportion (57.64%). This finding emphasizes the core mediating role of risk perception in the process of converting subversive cognition into governance outcomes. Lastly, the total effect of criticality cognition is significant, with both direct promotion of food safety governance and additional positive impacts through the mediator variable of risk perception. Specifically, the direct effect accounts for most of the proportion (72.14%), directly promoting the formation of food safety governance willingness; at the same time, the indirect effect through risk perception also significantly exists (27.86%), further enhancing the governance effect.

(3) Moderating Effect

The data in Table 6 reveal that the interaction term between risk perception and risk communication has a significant positive impact on food safety governance willingness, with a β coefficient of 0.0792 (*p* < 0.001); i.e., research hypothesis H5 is established. Further analysis showed that the change in the level of risk communication has a significant moderating effect on the impact of novel recognition, disruptive recognition, and critical recognition. Specifically, when the level of risk communication increases from one standard deviation below the mean (M−1SD) to one standard deviation above the mean (M+1SD), the effect value of novel recognition on food safety governance willingness increases from 0.252 to 0.283, the effect value of disruptive recognition increases from 0.0574 to 0.0948, and the effect value of critical recognition increases from 0.0542 to 0.108. These changes clearly demonstrate that as the level of risk communication increases, the positive impact of the cognitive path on food safety governance willingness is also enhanced, thereby confirming the moderating effect of risk communication between risk perception and food safety governance willingness. This means that under higher levels of risk communication, the impact of the cognitive path on food safety governance willingness will be more significant.

## 5. Conclusions and Implications

### 5.1. Research Conclusions

This study empirically analyzed the influence mechanism of cognitive perceptions of food safety events, risk perception, and risk communication on the public’s willingness to participate in food safety governance, validating the mediating role of risk perception between cognitive perceptions of food safety events and the public’s governance willingness, which aligns with the SOR model’s theoretical expectations regarding how individuals respond to external stimuli. However, the moderating role of risk communication was found to be more complex than anticipated and may vary across different socio-cultural contexts, indicating the need for further localization adjustments to the theory. The following specific details were found:

- The public’s novel, disruptive, and critical recognition of food safety events significantly influences their governance willingness, indicating that enhancing the breadth and depth of the public’s cognition of these events is key to stimulating their enthusiasm for participating in food safety governance;
- Risk perception plays a significant mediating role between cognitive perceptions of food safety events and governance willingness. The stronger the public’s risk perception of food safety events, the higher their willingness to participate in governance, emphasizing the centrality of enhancing public risk perception in improving the effectiveness of food safety governance;
- Risk communication significantly moderates the relationship between risk perception and food safety governance willingness. Effective risk communication strategies can enhance public cognition and risk perception of food safety events, thereby increasing their governance willingness, indicating that optimizing risk communication strategies is an important pathway to improving the efficacy of food safety governance.

### 5.2. Policy Recommendations

Firstly, in light of the importance of public cognition of food safety events, governments and relevant departments should increase publicity efforts on food safety incidents. Non-governmental organizations (NGOs) play a crucial bridging role in food safety governance. They can assist the government and enterprises in conducting food safety education and awareness campaigns to enhance public awareness of food safety. Additionally, NGOs can provide independent oversight and evaluation to ensure strict adherence to food safety standards, thereby increasing public trust in food safety. Through multi-channel and multi-form education and publicity, they should raise public awareness of food safety events, enhance the level of concern and attention to food safety issues, and thereby increase the willingness to participate in governance.

Secondly, to strengthen the public’s risk perception capabilities, governments and relevant departments should promptly and accurately communicate information about food safety events to the public, including the nature of the incident, the scope of impact, and potential risks. They should also encourage public participation in food safety supervision and reporting activities, empowering individuals to enhance their risk perception and sense of responsibility through action, making them more attentive to food safety issues and more engaged in governance.

Furthermore, governments and relevant departments should develop scientific and rational risk communication strategies to ensure that food safety information is conveyed to the public in a timely and accurate manner. Utilizing modern media channels such as the internet and mobile applications can improve the efficiency and the reach of risk communication, allowing more people to understand food safety events and take appropriate preventive measures.

Lastly, governments should enhance communication and collaboration with the public, businesses, and the media in participating in food safety governance activities. Encouraging public involvement in volunteer services, community supervision, and other food safety governance activities can foster a positive atmosphere where the entire society pays attention to and participates in food safety. Additionally, governments should strengthen the regulation and guidance of businesses and media to ensure they actively fulfill their social responsibilities and jointly safeguard food safety.

### 5.3. Limitations and Prospects

Despite the valuable insights gained from this study, several limitations should be acknowledged. First, the representativeness of the sample could be improved. Future research should consider expanding the sample size and diversity to enhance the generalizability and reliability of the findings. Second, while this study primarily examined the impact of food safety incidents on the public’s willingness to participate in food safety governance, numerous other factors may influence this willingness. Future studies could investigate additional influencing factors and their mechanisms, providing a more comprehensive understanding of public engagement in food safety governance. For example, factors such as social trust, cultural background, personal experiences, and media usage habits may also significantly influence the public’s willingness to engage in food safety governance. The level of trust that the public has in government, businesses, and media can affect their willingness to engage in food safety governance. If the public perceives these institutions as unreliable, they may be less inclined to participate in governance activities. Perceptions and responses to food safety issues may vary across different cultural backgrounds. For example, in some cultures, families and communities play a more significant role in food safety matters. Personal experiences could be another key factor. Whether individuals have personal experiences with food safety issues, such as food poisoning or purchasing substandard products, can significantly impact their willingness to participate in governance. For the “media usage habits”, the primary channels through which the public obtains food safety information, such as traditional media and social media, can also influence their perception of food safety incidents and governance willingness.

Future studies could investigate additional influencing factors and their mechanisms, providing a more comprehensive understanding of public engagement in food safety governance. These enhancements will not only strengthen the existing research but also contribute to more effective strategies for promoting public participation in food safety governance.

## Figures and Tables

**Table 1:** Sample Statistics.

Variable	Category	Sample Size (n)	Percentage (%)	Variable	Category	Sample Size (n)	Percentage (%)
Gender	Male	342	47.77	Marital status	Unmarried	113	15.78
	Female	374	52.23		Married	374	52.23
Age	Under 25	76	10.61		Divorced	102	14.25
	25–35	194	27.09		Widowed	40	5.59
	36–45	166	23.18		Remarried	87	12.15
	46–55	124	17.32	Family Structure	Single-person household	86	12.01
	56–65	106	14.80		Nuclear family	235	32.82
	66 and above	52	7.26		Single-parent family	124	17.32
Education level	Illiterate or primary school	49	6.84		Extended family	271	37.85
	Junior high school or below	82	11.45	Family income level	<5000 yuan	73	10.20
	High school/technical secondary/vocational school	185	25.84		7000–7999 yuan	89	12.43
	Associate degree	174	24.30		8000–11,999 yuan	167	23.32
	Bachelor’s degree	152	21.23		12,000–14,999 yuan	175	24.44
	Graduate degree or above	74	10.34		15,000–19,999 yuan	118	16.48
Occupation	Service industry personnel	196	27.37		>20,000 yuan	94	13.13
	Production and transportation workers	154	21.51	Residential area	Residential community	228	31.84
	Junior management and technical staff	121	16.90		Urban village	135	18.85
	Personnel in government, enterprises, and institutions	97	13.55		Peri-urban area	147	20.53
	Individual business owners	63	8.80		Staff dormitory	134	18.72
	Other	85	11.87		Frequently relocating	72	10.06

**Table 2:** Reliability test of variables.

Variable	Item	Parameters of Significance Test				Squared Multiple Correlation (SMC)	Composite Reliability	Average Variance Extracted (AVE)
		Estimate	SE	Est. SE	*p*-Value			
Novel recognition	NR1	0.876	0.019	46.182	***	0.784	0.909	0.715
	NR2	0.831	0.021	41.158	***	0.705		
	NR3	0.852	0.018	44.527	***	0.782		
	NR4	0.823	0.025	37.218	***	0.683		
Disruptive recognition	DR1	0.921	0.023	29.306	***	0.791	0.901	0.752
	DR2	0.872	0.035	23.217	***	0.831		
	DR3	0.804	0.031	22.452	***	0.775		
Critical recognition	CR1	0.723	0.037	21.543	***	0.645	0.793	0.565
	CR2	0.885	0.035	23.916	***	0.814		
	CR3	0.624	0.037	14.063	***	0.313		
Risk perception	RP1	0.913	0.012	82.625	***	0.862	0.902	0.654
	RP2	0.892	0.014	71.653	***	0.854		
	RP3	0.908	0.013	78.612	***	0.871		
	RP4	0.672	0.031	12.746	***	0.386		
	RP5	0.605	0.032	15.932	***	0.385		
Risk communication	RC1	0.793	0.028	31.869	***	0.672	0.868	0.689
	RC2	0.927	0.021	49.012	***	0.879		
	RC3	0.761	0.027	29.327	***	0.652		
Willingness to govern food safety	GW1	0.835	0.019	37.432	***	0.614	0.916	0.784
	GW2	0.962	0.011	67.231	***	0.963		
	GW3	0.854	0.019	43.562	***	0.761		

Note: *** *p* < 0.001, Abbreviations: NR—novel recognition; DR—disruptive recognition; CR—critical recognition; RP—risk perception; RC—risk communication; GW—willingness to govern food safety.

**Table 3:** Discriminant validity test.

Variable	Mean (M)	Standard Deviation (SD)	Novel Perception	Disruptive Perception	Critical Perception	Risk Perception	Risk Communication	Willingness to Govern Food Safety
Novel recognition	2.251	0.947	0.846					
Disruptive recognition	6.287	0.851	0.353 **	0.867				
Critical perception	5.414	0.819	0.316 **	0.372 **	0.752			
Risk perception	5.186	1.042	0.197 **	0.253 **	0.345 **	0.809		
Risk communication	5.261	1.193	0.376 **	0.249 **	0.412 **	0.387 **	0.830	
Willingness to govern food safety	4.761	1.286	0.254 **	0.006 **	0.352 **	0.373 **	0.257 **	0.854

Note: ** *p* < 0.01, M (Mean) indicates the arithmetic mean of all items in the dimension; the numbers on the diagonal are the square roots of the average variance extracted (AVE).

**Table 4:** Hypothesis Testing.

Variable	Risk Perception			Willingness to Govern Food Safety		
	*β*	*SE*	*p*	*β*	*SE*	*p*
Novel recognition	0.1740 ***	0.0547	0.0003	0.1670 **	0.0528	0.0031
Disruptive recognition	0.2980 ***	0.0523	0	0.0735	0.0424	0.0714
Critical recognition	0.2090 ***	0.0541	0.0014	0.3450 ***	0.0527	0
Risk perception				0.2540 ***	0.0603	0
Risk communication				0.1250 ***	0.0572	0.0026
Perception × communication				0.0792 ***	0.0436	0.0004
R2	0.1360			0.1820		
*F* value	14.2000			14.9000		

Note: ** *p* < 0.01; *** *p* < 0.001.

**Table 5:** Bootstrap results for mediation effects.

Effect Type	Effect Size	SE Value	Bootstrap 95% CI		Relative Effect Proportion (%)
			LLCI	ULCI	
Independent variable: Novel recognition					
Total effect	0.2760	0.0581	0.1600	0.4310	100
Direct effect	0.1810	0.0524	0.1190	0.2080	65.4300
Indirect effect	0.0955	0.0236	0.0038	0.1720	34.5700
Independent variable: Disruptive recognition					
Total effect	0.1870	0.0582	0.1230	0.2610	100
Direct effect	0.0793	0.0527	0.0041	0.1240	42.3600
Indirect effect	0.1080	0.0234	0.0054	0.1430	57.6400
Independent variable: Critical recognition					
Total effect	0.3520	0.0514	0.2080	0.4060	100
Direct effect	0.2540	0.0542	0.1050	0.3830	72.1400
Indirect effect	0.0980	0.0246	0.0465	0.1390	27.8600

Notes: SE = Standard error value; CI = Confidence interval; LLCI = Lower limit of the confidence interval; ULCI = Upper limit of the confidence interval.

**Table 6:** Mediating moderating effect of risk communication.

Path	Risk Communication	Effect Size	SE Value	LLCI	ULCI
Novel recognition → Risk communication → Willingness to govern food safety	M−1SD	0.2520	0.0713	0.0523	0.4180
	M	0.2670	0.0614	0.1570	0.2940
	M+1SD	0.2830	0.0655	0.2440	0.5180
The mediated moderating effect		0.0153	0.0129	0.0042	0.0264
Disruptive recognition → Risk communication → Willingness to govern food safety	M−1SD	0.0574	0.0315	0.0147	0.0941
	M	0.0761	0.0302	0.0134	0.1910
	M+1SD	0.0948	0.0294	0.0362	0.1650
The mediated moderating effect		0.0187	0.0134	0.0034	0.0462
Critical recognition → Risk communication → Willingness to govern food safety	M−1SD	0.0542	0.0291	0.0105	0.2020
	M	0.0813	0.0264	0.0433	0.1550
	M+1SD	0.1080	0.0365	0.0472	0.2080
The mediated moderating effect		0.0271	0.0172	0.0013	0.0632

M−1SD = Mean minus one standard deviation; M = Mean; M+1SD = Mean plus one standard deviation.

## Data Availability

The original contributions presented in the study are included in the article, further inquiries can be directed to the corresponding author.
